# A short review of the sustainable utilization of coal gangue in environmental applications

**DOI:** 10.1039/d4ra06071g

**Published:** 2024-12-12

**Authors:** Lihui Gao, Yanhong Liu, Keyi Xu, Lu Bai, Nan Guo, Shulei Li

**Affiliations:** a State Key Laboratory of Water Resource Protection and Utilization in Coal Mining Beijing 102211 China lu.bai.a@chnenergy.com.cn; b School of Environment Science and Spatial Informatics, China University of Mining and Technology Xuzhou 221116 China; c National Institute of Clean-and-Low-Carbon Energy (NICE) Beijing 102211 China; d National Engineering Research Center of Coal Preparation and Purification, China University of Mining and Technology Xuzhou 221116 China

## Abstract

The production of coal gangue, a by-product of coal mining and washing, is rapidly increasing due to growing energy consumption. As the accumulated coal gangue has not been appropriately utilized, this has resulted in a squander of resources, waste disposal problems, and environmental pollution issues. However, coal gangue, a form of solid waste, exhibits various potential applications in the field of recycling. Currently, the processing capacity and utilization rate of coal gangue are insufficient to meet the demands of global low-carbon environmental protection. As such, this paper provides a detailed overview of the physical and chemical properties of coal gangue and its resource utilization methods in the environmental field, particularly in soil remediation, soil reclamation, the preparation of adsorbents for heavy metals, dyes, and CO_2_, and as catalysts for organic degradation and catalytic cracking. This review highlights the significance of coal gangue classification according to its properties for the comprehensive utilization of gangue. Additionally, we briefly introduce and discuss suggestions for future directions of research and development in this area. Nevertheless, with regard to the environmental impact of secondary pollution and the efficiency of coal gangue utilization, there remain numerous obstacles that hinder future research. This review is expected to provide significant insights into the development of coal gangue utilization strategies, potentially facilitating the efficient and practical large-scale management of solid waste.

## Abstract 2

This review summarizes the environmental applications of coal gangue and highlights the challenges and future directions of its utilization as a large-scale resource.

## Graphical abstract

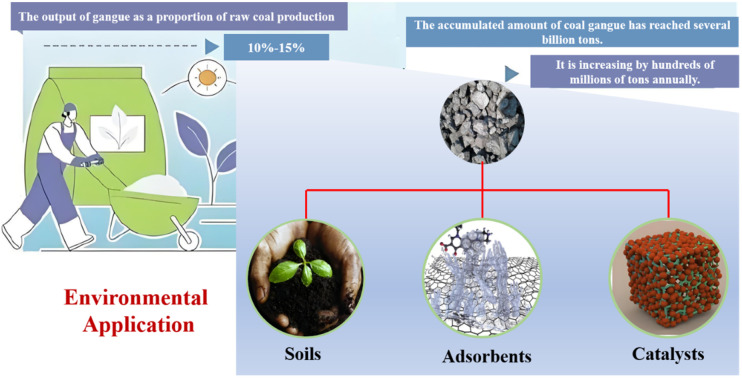

## 1. Introduction

Coal gangue is one of the largest industrial solid wastes discharged during coal mining and processing, and has a lower carbon content and higher hardness than coal. Due to its low calorific value and it being difficult to utilize, large amounts of coal gangue are piled on the mine surface, occupying land, causing soil erosion, landslides, debris flows, and other geological disasters, all of which greatly affect the surrounding ecological safety.^1^ China is the largest producer of coal gangue globally, with an accumulated production exceeding 5 billion tons. The production rate continues to rise annually by 300–350 million tons. By 2020, the total production of coal gangue in China is expected to reach approximately 729 million tons, posing a significant environmental concern.^2^

Coal gangue contains a significant amount of metallic and non-metallic elements. Its accumulation not only occupies extensive land resources but also leads to serious environmental problems. Heat gradually accumulates during the coal gangue accumulation process, leading to its oxidation and spontaneous combustion,^3,4^ which releases a large number of harmful gases such as SO_2_, NO_*x*_, and CO into the atmosphere. Additionally, when stored in the open, coal gangue is subjected to natural conditions such as sunlight, rain, and wind, which can cause toxic and harmful elements like cadmium and mercury to leach into surface water or soil through rainwater infiltration, and subsequently into shallow groundwater through soil infiltration, severely affecting the environment.^5^ Therefore, realizing the large-scale utilization of coal gangue as a resource is an urgent task required to mitigate its negative impacts on the ecological environment.

Over the past few decades, the utilization of coal gangue has gained attraction among researchers from various countries.^2^ Each country exhibits different approaches to utilize coal gangue. For example, in the United Kingdom, coal gangue is primarily used for road filling, dams, and other civil engineering projects, as well as for preparing low-grade concrete. Germany, on the other hand, utilizes coal gangue as a raw material in coal-fired power plants for electricity generation.^6,7^ China, as the world's largest country in terms of coal gangue production, has conducted extensive research on its resource utilization.^8^ The comprehensive utilization of coal gangue can be divided into two approaches. In one approach it is directly mixed with other solid waste, which is a simple process, a mature technology, easy to industrialize, and results in a large consumption of the gangue. The gangue is primarily used in the fields of building materials, agricultural production, green cover and backfilling of underground mining areas.^9–13^ It is the most widely used method at present. The second approach focuses on unlocking the high-value potential of coal gangue through advances applications. This includes utilizing gangue for power generation, extracting valuable elements, and synthesizing high value-added products like zeolites, adsorbents, flocculants, ceramics, and fiber reinforcing agents.^14–16^ Despite significant research achievements in coal gangue resource utilization technologies in China, the overall utilization volume remains inadequate compared to its production yield. For instance, in 2021, China produced 743 million tons of coal gangue, yet only 543 million tons were utilized. Addressing this gap between coal gangue production and utilization shows an opportunity for future research and development in maximizing the value extracted from coal gangue resources.

Therefore, the thorough utilization of coal gangue is essential for advancing sustainable coal extraction, expanding the coal industry value chain, fostering a resource and environmentally friendly society, and facilitating the shift towards a more sustainable economic model. In light of this, there is a necessity to review and synthesize the knowledge accumulated to date on the multifaceted comprehensive utilization of coal gangue, in order to explore the trends and potential of applying it more extensively.

## 2 Coal gangue characterization

Coal gangue is a black-gray sedimentary rock associated with coal seams, composed of a variety of rock and mineral compositions. It is characterized by low carbon content, higher hardness compared to coal, and a dry basis ash content greater than 50%. The mineral composition of coal gangue is more complicated in terms of mineral components, including kaolinite, illite, quartz, montmorillonite, limestone, feldspar, dolomite, rhodochrosite, and pyrite, *etc.*Table 1 shows the mineral compositions of coal gangue from different countries. It can be seen that, compared to other countries, coal gangue in China is rich in kaolinite and has a moderate quartz content.^17,18^ This indicates that coal gangue has significant potential for application in the environmental field, particularly in the synthesis of adsorbents and catalysts. Chemically, coal gangue is a mixture of organic and inorganic substances. The major components of gangue are SiO_2_ and Al_2_O_3_, comprising approximately 60–90%. Coal gangue additionally encompasses a variety of trace elements such as Fe, Ca, Mg, Mn, Cu, Zn, Cl, P, and S along with their respective oxides. Furthermore, it contains minor quantities of less common metals like Ti, V, Co, Ga and other elements,^7,19^ as well as organic compounds including hydrocarbons and humus.^20^ Coal gangue can be classified into four categories based on the composition of Si and Al, such as carbonaceous claystone gangue, sandstone and siltstone type gangue, calcareous rock gangue and high alumina gangue.^21^ Carbonaceous claystone gangue contains 24–56% SiO_2_ and 14–34% Al_2_O_3_, and is primarily utilized for combustion power generation. Sandstone and siltstone coal gangue are characterized by a high silicon content and the presence of minerals such as quartz, feldspar, and mica, and a SiO_2_ content ranging from 53–88%. Sandstone and siltstone coal gangue are commonly used in the preparation of building materials like cement. Calcareous rock gangue is characterized by low to moderate amounts of Si, high levels of Ca, and the presence of minerals such as calcite, dolomite, and rhodochrosite. The SiO_2_ content ranges from 30–40%, while the CaO content is significantly higher compared to other types of coal gangue, at approximately 10–45%. High-aluminum coal gangue is characterized by high Al content, moderately high Si, low K, low Ca, low Mg, low Fe, and low Na. It contains 42–54% SiO_2_ and 37–44% Al_2_O_3_, making it a high-quality raw material for the preparation of adsorbents and the recovery of iron, aluminum minerals, and SiO_2_.

**Table 1:** Mineralogical compositions of coal gangues in various countries^2,7^

Mineral	Belgium	Czech Republic	Germany	Spain	Britain	Russia	China
Illite	80	10–45	41–66	20–60	10–31	5–30	10–30
Kaolinite	12	20–45	4–25	3–30	10–40	1–60	10–67
Chlorite	5	0–15	1–3	0–7	2–7		2–11
Quartz	8	10–50	13–27	5–57	15–25		15–35
Iron ore	0.5	0–25	0.5–5		2–10	0.2–8	2–10
Organic matters	10	0–25	5–10	4–30	5–25	8–40	5–25

The calorific value of coal gangue denotes the energy released upon its complete combustion per unit mass under specific conditions. Generally, the calorific value of coal gangue decreases with increasing ash content, and increases with increasing carbon content and volatile matter. Table 2 illustrates the applications of coal gangue at varying carbon content/calorific values. From Table 2, it can be observed that coal gangue with a calorific value in the range of 6270–12550 kJ kg^−1^ is commonly applied to power generation and fuels, while its calorific value below 2090 kJ kg^−1^ usually could be used as a raw material in cement filler, landfilling and other building materials.^7^ The meltability of coal gangue refers to the process where it undergoes softening and melting at elevated temperatures under specific conditions. Due to its high SiO_2_ and Al_2_O_3_ content, the ash melting point of coal gangue is pretty high, ranging from approximately 1050 °C to 1800 °C. Additionally, coal gangue, characterized by its low purity, low density (from 1.0 to 2.5 g cm^−3^), high hardness and strength, also possesses certain properties such as expandability, plasticity, and shrinkage, which endow it with characteristics of fire resistance, acid resistance, and low ion exchange capacity.^22^ Based on these qualities, coal gangue can be utilized in refractory materials, building materials, catalyst carriers, and for various other applications.

**Table 2:** Industrial utilization of coal gangue

Type	Carbon content	*Q* (kJ kg^−1^)	Utilization routes
IV	>20%	6270–12 550	Power generation, fuels
III	6–20%	2090–6270	Building materials
II	4–6%	<2090	Cement filler, landfilling, paving, back filling
I	<4%	<2090	Cement filler, landfilling, paving, back filling

## 3 Environmental applications of coal gangue

**Fig. 1:**
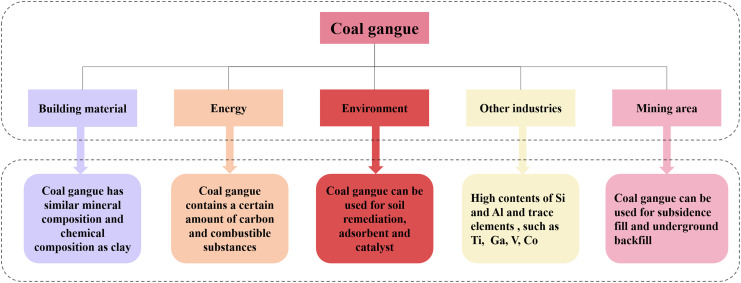
The current fields in which coal gangue is used.

With the increasing production and therefore accumulation of coal gangue and the tightening of environmental regulations, the efficient and rational utilization of coal gangue has become an inevitable choice for the development of an environmentally friendly society. As shown in Fig. 1, coal gangue is widely used in building materials, the energy industry, and in environmental and other applications. The utilization of recycled coal gangue is recognized as an eco-friendly solution that not only addresses its disposal effectively but also promotes cost-effective material use and the sustainable management of resources.

### 3.1 Application in developing soil quality

#### 3.1.1 Soil remediation

In the context of contemporary environmental protection and sustainable development, soil remediation has become a critical issue. Soil pollution, particularly due to industrial activities and improper waste disposal, which results in heavy metal and organic contamination, poses severe threats to ecosystems and human health.^23^ Against this backdrop, coal gangue has garnered extensive attention as a potential soil remediation material. Coal gangue contains a high level of organic matter, making it an ideal substrate for carrying nitrogen, phosphorus, and potassium. The scientific and rational addition of coal gangue to soil has the potential to reduce soil adhesion and improve soil porosity.^24^ The pore structure of soil with added coal gangue enhances the dissolution of ore fertilizers and oxygen in water. This process improves the absorption of nutrients and respiration of plant roots, ultimately benefiting plant growth and health. Du *et al.*^25^ explored the use of coal gangue as a novel planting substrate component to address the growing shortage of land resources. Their results indicated that coal gangue significantly improved both the chemical properties (organic matter, total N, total P, available N, available P, and available K) and the physical properties (bulk density, total porosity, capillary porosity, soil moisture content, and permeability coefficient) of the substrate.

**Fig. 2:**
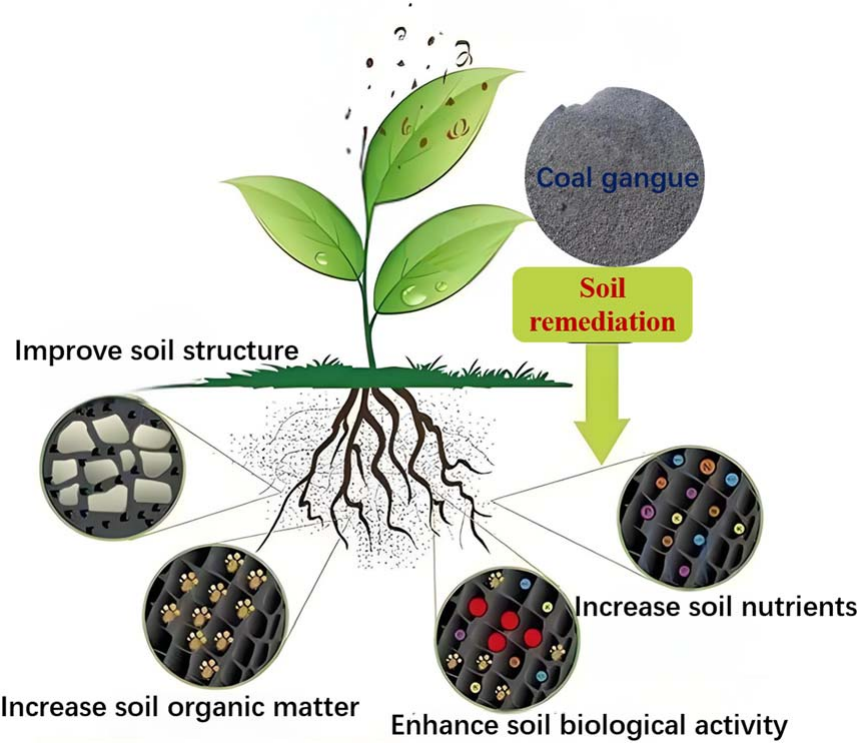
The role of coal gangue in soil remediation.

Moreover, as shown in Fig. 2, the addition of coal gangue into the soil structure not only improves the properties of the soil matrix but also fosters a favorable environment for microorganisms, enhancing the metabolism of aerobic and facultative bacteria and significantly increasing soil microbial biomass, thus promoting plant growth.^26^ For example, due to the oxidation of organic sulfur with the help of microorganisms, high-sulfur coal gangue effectively lowers soil pH and Eh values, thereby improving the crop growth environment.^24,27^ Wang *et al.*^28^ used high-sulfur coal gangue to amend soda-saline soils. Results showed that the treatment with 70-mesh high-sulfur coal gangue was most effective, and with application rates of 16, 32, 48, and 64 g kg^−1^, the pH of the saline-alkali soil decreased by 6.66%, 11.20%, 14.67%, and 16.70%, respectively. The exchangeable sodium percentage (ESP) decreased by 59.96%, 68.24%, 69.81%, and 73.76%, and the exchangeable sodium ion content decreased by 19.05%, 21.09%, 17.69%, and 19.05%, demonstrating that high-sulfur coal gangue effectively ameliorates soda-saline soils. Thus, using coal gangue as a raw material for soil remediation not only improves soil structure but also significantly enhances soil biological activity, increases soil organic matter, and boosts nutrient levels.

However, coal gangue has potential advantages in soil improvement, but its poor water retention limits its capability as a high-quality soil amendment. Similarly, sewage sludge, another massively produced type of waste, contains abundant organic matter, nitrogen, and other nutrients, along with diverse microbial communities that meet plant growth needs. Currently, research favorably regards coupled synergistic soil improvement using coal gangue and sewage sludge. Table 3 summarizes recent applications of sludge and coal gangue in soil remediation, demonstrating the feasibility of using sludge coupled with coal gangue to remediate polluted soils. Coal gangue has a higher bulk density than typical soil and fewer capillary pores; its incorporation into soil enhances soil aeration and porosity, providing certain nutritional components but struggling to form soil aggregates and lacking active microbes. Sludge, when used in agriculture, has poor air permeability but increases levels of nitrogen and phosphorus, organic matter content, microbes in the soil, it adjusts the soil pores, improves the soil granular structure, and when used with coal gangue achieves the complementary advantages of both waste types. This approach facilitates their respective strengths and complementary benefits. However, coal gangue and sludge contain harmful substances such as heavy metals, and the concentration of these metals might increase over time due to leaching. Although coal gangue can be applied to soil remediation, the potential secondary pollution it may cause should also be given due attention.

**Table 3:** Utilization of sludge and coal gangue for soil remediation

Research objects	Research method	Main finding
Sewage sludge and coal gangue	A mixture and blending method, verified using a tall fescue pot experiment	Sludge and coal gangue can significantly increase the clay percentage in the substrate, which is beneficial for water and nutrient retention^29^
Sewage sludge and coal gangue	Modification of sludge and coal gangue composite substrates using ZnCl_2_ and hydrochloric acid	The composite activated carbon prepared from sludge and coal gangue exhibits a well-developed porous surface structure and an increased variety of functional groups, which enrich the dominant bacterial species^30^
Sewage sludge and coal gangue	Mixture blending and leaching pot experiment	It can effectively immobilize heavy metal elements in coal gangue, reducing the leaching of heavy metal content from coal gangue^31^
Sewage sludge, coal gangue and soil	Pot experiment with sewage sludge, coal gangue and mixed soil substrates	The plant–soil system can gradually reduce the concentration of harmful substances in the growth medium^32^
Sewage sludge, coal gangue, fly ash and soil	A pot experiment was conducted by mixing sludge, coal gangue, and fly ash into the soil	This is beneficial for plant growth, and the level of heavy metal contamination in the composite substrate remains the same^33^
Sewage sludge, coal gangue and soil	A pot experiment was conducted by mixing differently treated sludge and coal gangue into the soil	This can promote the growth of the underground parts of certain plants^34^
Sewage sludge, coal gangue, fly ash and soil	A pot experiment was conducted by mixing sludge, coal gangue, and fly ash into the soil	The nutrient composition of the soil has been improved^35^
Sewage sludge, coal gangue, fly ash and soil	A terraced experiment was conducted by mixing sludge, coal gangue, and fly ash into the soil	Increases the fertility of the soil in coal gangue hill reclamation^36^

#### 3.1.2 Soil reclamation

Coal mining contributes to rapid economic growth but also leads to significant ecological and environmental issues. In China, the total mine subsidence area has reached 1.35 million hectares and is expanding by 70 000 hectares annually, the highest rate globally.^37^ Subsidence creates permanent basins, severely disrupting soil properties, hindering nutrient cycling, and limiting plant growth.^38^ Reclamation efforts generally involve landfill techniques, including the use of coal gangue, ash, or sediments from rivers and lakes to fill the subsidence areas. Among these materials, coal gangue stands out as the most widely used filling material due to several advantages: (a) it is a solid waste from mining, accounting for approximately 10–20% of coal production, providing a substantial and readily available source of filling material;^39^ (b) its local availability reduces transportation costs; (c) with a utilization rate of less than 15%, there is an accumulation of 3.8 billion tons of coal gangue, increasing by 200 million tons annually,^37^ and thus its use can mitigate the covering and occupation of land by stockpiled gangue;^40^ (d) utilizing coal gangue can alleviate environmental pollution caused by coal gangue dumps;^41^ and (e) its appropriate hydraulic conductivity, sorption characteristics, and leaching behavior make it suitable for reuse as a landfill material.^42^

However, the reclamation rate for mining subsidence remains around 35%.^37^ A key challenge is the typically low productivity of reclaimed soil, which complicates efforts to restore it to its original condition within a short timeframe. Consequently, numerous researchers have investigated the quality progression and spatiotemporal changes of reclaimed soil in coal mine subsidence areas from various perspectives.^43,44^ Kumar *et al.* examined variations in soil organic carbon (SOC) and glomalin-related soil protein (GRSP) fractions, along with other critical physicochemical, biochemical, and microbial soil quality variables, in relation to revegetation over a chrono-sequence of reclaimed mine spoils (1 to 26 years) in a tropical region.^43^ Similarly, Li *et al.* conducted long-term monitoring of the physicochemical properties in coal gangue-reclaimed subsidence areas. Their results indicated that after 10 years of reconstruction, the soil variables had nearly reverted to their pre-damage states. However, the reclaimed soil exhibited increased fragility due to stronger inter-variable correlations.^44^

**Fig. 3:**
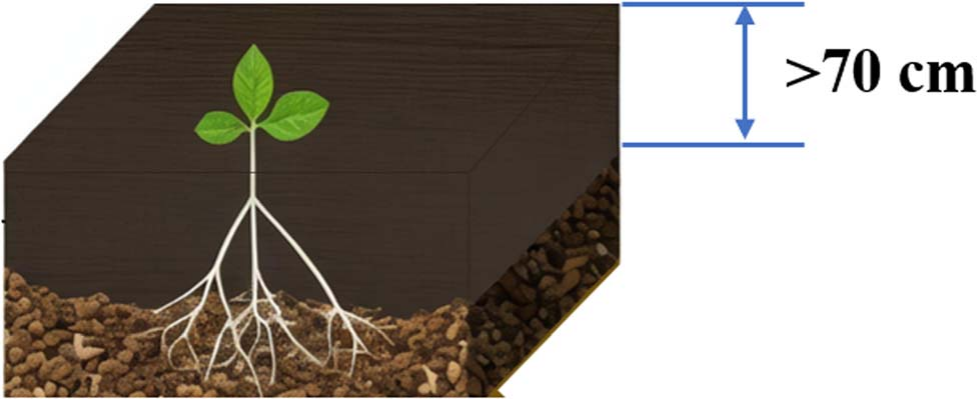
Schematic diagram of a coal gangue-filled reclamation profile.

In addition, the thickness of the soil cover over the gangue substrate significantly affects crop growth. When the soil cover thickness exceeds 70 cm, the crop yields approach that of local farmland, as shown in Fig. 3. However, when the cover thickness is less than 70 cm, crop growth is adversely affected, particularly at 30 cm, where crops are prone to wilting and water deficiency.^45^ Some studies have shown that interlayered profiles can improve the water and fertilizer retention of the soil profile.^37,46,47^ However, whether covering coal gangue with soil or using an interlayered structure with coal gangue and soil, a soil thickness of at least 70 cm is required to minimize the adverse effects of coal gangue.^37,45^

In summary, the utilization of coal gangue in soil application holds significant potential. However, when selecting the raw material for soil remediation/reclamation, it is crucial to consider the chemical elements present in different types of coal gangue. This involves not only adhering to industry standards to control toxic and hazardous substances but also finding the most effective strategies for coal gangue utilization to achieve the best outcomes.

### 3.2 Application in developing adsorbents

The development of low-cost and environmentally friendly adsorbents derived from waste coal gangue represents an innovative approach to eliminate industrial wastewater pollution, promote cleaner production, and mitigate the greenhouse effect. Coal gangue primarily consists of clay minerals and has a porous structure, and its main component is the mineral kaolinite. Kaolinite, a 1 : 1 type silicate clay mineral, consists of silicon–oxygen tetrahedral sheets and aluminum–oxygen octahedral sheets, and features active hydroxyl groups on its surface, strong polarity, well-developed pores, and a large specific surface area. Moreover, the Si–O–Si bonds linking the tetrahedral and octahedral layers can be cleaved under the activation environment, resulting in the decomposition of kaolinite into reactive silicon and aluminum species. These structural units can be reassembled in specific crystallization environments to generate novel silicate materials with varied structures and enhanced properties.^48^ Based on the characteristics mentioned above, coal gangue and its modified or derivative materials exhibit a porous structure with polar siloxane bonds, which enhance the capacities to physically and chemically adsorb pollutant molecules.^49^ Currently, the applications of porous materials prepared from coal gangue mainly focus on the adsorption of heavy metal ions, organic dyes, and CO_2_.

#### 3.2.1 Adsorption of heavy metals

Heavy metal pollution is one of the three major forms of water contamination globally. Even small amounts of heavy metals can cause significant harm, as they do not degrade but instead accumulate or transform, posing serious threats to the environment and human health. Materials (such as zeolites^50–53^ and their composites,^54–56^ porous coal gangue microspheres,^57^ porous geopolymers,^58^ and porous carbon materials^59^) derived from coal gangue, have achieved notable success in the adsorption of heavy metals.

**Fig. 4:**
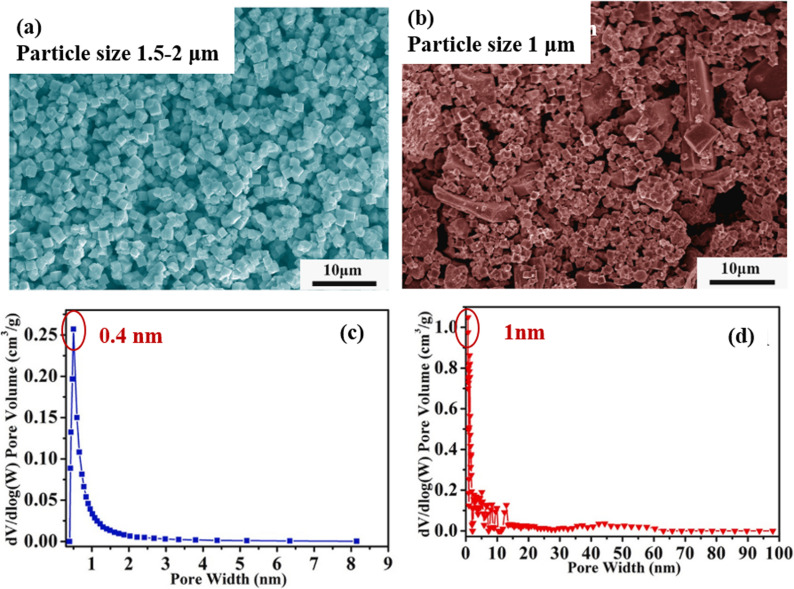
SEM images of (a) a pure zeolite derived from coal gangue and (b) a zeolite and bamboo activated carbon composite. Pore size distribution curves of (c) a pure zeolite derived from coal gangue and (d) a zeolite and bamboo activated carbon composite.^55^

Table 4 presents the heavy metal adsorption performance of various coal gangue-based modified materials. It can be observed that various metal ions such as Cu^2+^, Co^2+^, Cd^2+^, Pb^2+^ and Zn^2+^ could all be adsorbed effectively by coal gangue-derived porous materials. In addition, coal gangue modified materials coupled with carbon materials (such as coal, bamboo, *etc.*) exhibit superior heavy metal ion adsorption performance compared to coal gangue modified materials alone. Wang *et al.* synthesized a hollow sphere adsorbent from waste coal gangue through a spray drying-calcination process. The maximum adsorption capacities of the adsorbent for Cu^2+^ and Pb^2+^ at 25 °C were calculated to be 6.570 mg g^−1^ and 18.904 mg g^−1^, respectively.^57^ Lu *et al.* synthesized a NaX type zeolite from coal gangue and its adsorption capacity for copper ions (Cu^2+^) and cobalt ions (Co^2+^) were reported to be 45.05 mg g^−1^ and 44.53 mg g^−1^.^53^ In contrast, zeolite-activated carbon composites with a hierarchical porous structure and a specific surface area of up to 879.1 m^2^ g^−1^ were synthesized using coal gangue as the silicon and aluminum source and bamboo as the carbon source through a calcination–hydrothermal process. As shown in Fig. 4, the micropores on the zeolite can capture small-sized ionic heavy metal ions. The bamboo-based zeolite-activated carbon composite has a large specific surface area, and the zeolites grown on it have a smaller particle size, which allows the smaller-sized zeolites to capture heavy metal ions deeper within the pores of the activated carbon.^55^ In addition, compared to pure analcime, the coal–analcime composite synthesized by Jie *et al.* using coal and coal gangue as raw materials exhibited a higher specific surface area and a more extensive pore size distribution. Additionally, the maximum Pb^2+^ adsorption capacity reached 268 mg g^−1^.^56^

**Table 4:** Materials synthesized from coal gangue and their heavy metal adsorption performance

Material	Raw material	Heavy metal ion	Maximum adsorption capacity (mg g^−1^)	Ref.
NaX zeolite	Coal gangue	Cu^2+^	45.1	53
		Co^2+^	44.5	
NaX zeolite	Coal gangue	Cd^2+^	38.6	50
Coal gangue microspheres	Coal gangue	Cu^2+^	6.5	57
		Pb^2+^	18.9	
Hollow gangue microsphere/geopolymer	Hollow gangue microspheres, geopolymer matrix	Cu^2+^	17.5	58
		Cd^2+^	18.2	
		Zn^2+^	14.3	
		Pb^2+^	138.8	
Zeolite-activated carbon composite	Coal gangue, bamboo	Cu^2+^	104.9	55
Coal–analcime composite	Coal gangue, coal	Pb^2+^	268	56
Zeolite-activated carbon	Coal gangue, coal	Cu^2+^	111.3	54

#### 3.2.2 Adsorption of organic dyes

**Fig. 5:**
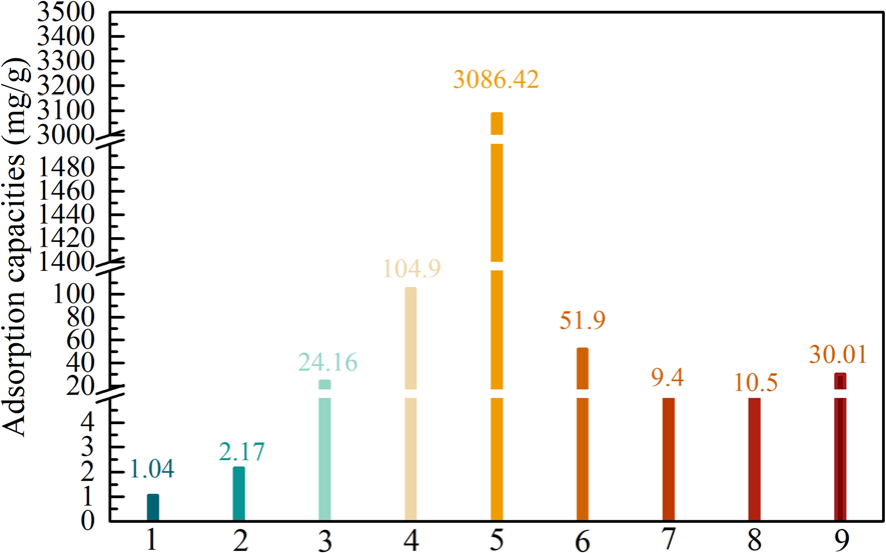
Organic dye adsorption capacities of various porous materials based on coal gangue, as reported in the literature. (1) Porous ceramic microspheres, basic fuchsine, (2) porous ceramic microspheres, cationic blue, (3) porous ceramic microspheres, cationic red, (4) porous zeolite/activated carbon composites, rhodamine B, (5) porous carbon, rhodamine B, (6) porous gangue/palygorskite composites, methylene blue, (7) porous activated material, methylene blue, (8) porous alkali-activated material, methylene blue, and (9) porous ceramic microspheres, methylene blue.

Adsorbent materials synthesized from coal gangue, such as porous ceramic microspheres, zeolite-activated carbon composites, not only exhibit good adsorption efficiency for heavy metal ions but also show excellent adsorption performance for organic compounds and dyes. As presented in Fig. 5, a porous adsorbent prepared from coal gangue exhibits good adsorption efficiency for various organic dyes, such as basic fuchsin, cationic blue, cationic red, MB *etc*. Zhou *et al.* demonstrated that ceramic adsorbents derived from coal gangue could adsorb 1.044 mg g^−1^ of cationic red X-5GN and 2.170 mg g^−1^ of cationic blue X-GRRL, achieving nearly 100% removal efficiency.^60^ Similarly, Li *et al.* showed that zeolite/activated carbon composites could adsorb rhodamine B with a capacity of 32.75 mg g^−1^ and a removal efficiency of 94.2%.^54^ Yan *et al.* reported that low-cost porous microspheres synthesized from coal gangue waste exhibited an adsorption capacity of 24.6 mg g^−1^ for methylene blue, with a removal rate of 98%.^61^

Although the aforementioned material can achieve the efficient removal of organic dyes, its adsorption capacity is generally low.

**Fig. 6:**
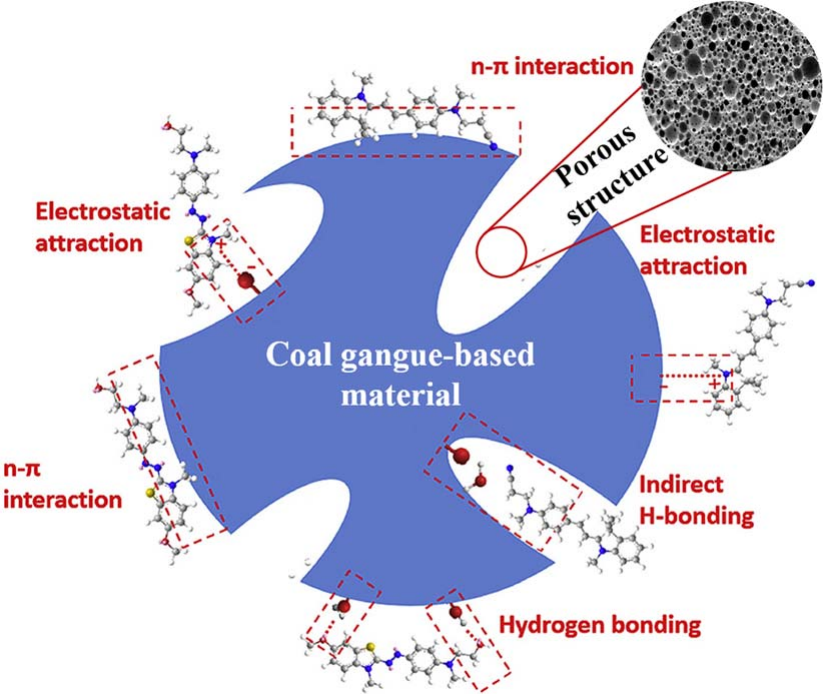
Schematic showing mechanisms by which gangue-based materials adsorb dyes.^60^

Therefore, some studies have attempted to convert coal gangue-based materials into porous silicates through activation to achieve high-capacity adsorption of dyes from wastewater. The porous silicate@carbon composites synthesized by Zhao *et al.* with a specific surface area of 650.96 m^2^ g^−1^ can achieve efficient adsorption of methylene blue and basic Red 14, with maximum adsorption capacities reaching 269.86 mg g^−1^ and 504.91 mg g^−1^, respectively.^48,62^ Wu *et al.* prepared a hierarchical porous carbon adsorbent with excellent adsorption performance from coal gangue using a two-step method, achieving an adsorption capacity of up to 3086.42 mg g^−1^ for rhodamine B.^59^ According to the reported articles, the mechanism by which the organic dyes are adsorbed onto the coal gangue-based porous materials likely involves the following interactions, as shown in Fig. 6.^48,60,62^ (1) Surface charge interactions: the incorporation of silico-aluminum isomorphous substituents or other metal ion substituents generates surface negative charges; this facilitates electrostatic interactions between the positively charged dye molecules and the negatively charged surfaces of the coal gangue-based materials. (2) n–π interactions: interactions between surface cationic metal ions (*e.g.*, Al^3+^, K^+^, Na^+^, Mg^2+^) and the aromatic or conjugated structures of the dye molecules may involve n–π interactions. (3) Hydrogen bonding: direct hydrogen bonding could occur between the hydrogen atoms of surface hydroxyl groups and the nitrogen, oxygen, or sulfur atoms within the dye molecules.

In summary, porous adsorbents derived from coal gangue can achieve efficient adsorption of organic dyes through electrostatic attractions, hydrogen bonding, and π–π interactions. However, current research primarily focuses on powder adsorption, including small particle-sized microspheres. However, powders are not ideal for adsorbent recovery without conductivity. Consequently, there is a need for increased research into the development of suitable fluidized beds, foams, porous monoliths, and granules, which hold greater engineering application value.

#### 3.2.3 Adsorption of carbon dioxide

The increased concentration of greenhouse gases like carbon dioxide in the atmosphere has been one of the most urgent global environmental concerns due to its climate-changing potential. Accordingly, the development of strategies towards the mitigation of CO_2_ and other green-house gases is of great importance. The primary methods for CO_2_ collection currently include carbon capture and storage (CCS) and carbon capture and utilization (CCU).^49^ Among the various CO_2_ capture techniques, adsorption using porous materials is particularly promising due to its flexibility, high automation capability and low energy penalty.^63–65^ Recently, porous materials derived from coal gangue, such as mesoporous magnesium silicate, low silica X zeolite *etc.*, have shown significant potential in mitigating greenhouse gas emissions. A novel mesoporous silica material (M-SiO_2_) with an MCM-41 structure was synthesized through a hydrothermal reaction using coal gangue as the raw material. M-SiO_2_ exhibited an adsorption capacity of 9.61 mg g^−1^ for CO_2_ at 25 °C and a CO_2_ concentration of 8%. To further enhance the CO_2_ adsorption performance, M-SiO_2_ was chemically modified with ethylenediamine (EDA), which significantly increased the CO_2_ adsorption capacity to 83.5 mg g^−1^ under the same conditions.^66^ In addition, the CO_2_ capture adsorbents synthesized from coal gangue are also highly selective for CO_2_. Ai *et al.* synthesized a mesoporous magnesium silicate from coal gangue by reacting it with magnesium chloride through stirring. The synthesized material exhibited a CO_2_ adsorption capacity of 1.02 mmol g^−1^ at 25 °C, which is 33 times higher than that of N_2_.^67^ LSX zeolite with a Si/Al ratio of 1.05 and specific surface area of 634 m^2^ g^−1^ was synthesized from low-grade coal gangue *via* a two-step activation method. The LSX exhibited an adsorption capacity of 4.99 mmol g^−1^ for CO_2_, and the selectivity coefficients for CO_2_ in binary mixed gases CO_2_/N_2_ and CO_2_/CH_4_ can reach 376 and 200, respectively.^68^ All these findings demonstrate that the adsorbents synthesized from coal gangue show tremendous potential for selectively adsorbing CO_2_.

**Fig. 7:**
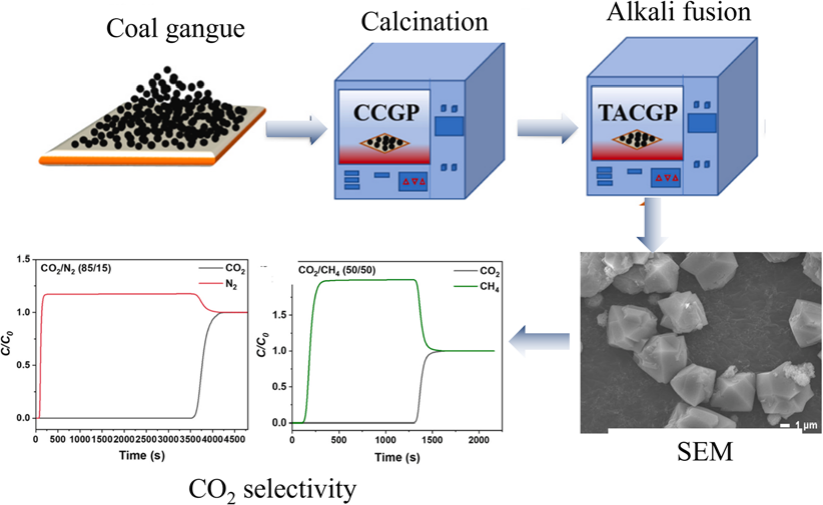
The process of synthesizing CO_2_ adsorbents from coal gangue and their CO_2_ adsorption performance.

However, the preparation of CO_2_ adsorbents from coal gangue typically requires high temperatures and strongly alkaline conditions. As shown in Fig. 7, after coal gangue was activated at 1200 °C, it is subjected to alkali fusion with NaOH at a mass ratio of 1 : 1.2 at 550 °C. Subsequently, by optimizing the addition of KOH and crystallization time, an X-type zeolite with high CO_2_ adsorption and selectivity is synthesized.^68^ Ai *et al.* extracted a silicon precursor for the preparation of mesoporous magnesium silicate for CO_2_ adsorption at a calcination temperature of 1000 °C, a NaOH concentration of 150 g L^−1^, and a mass ratio of 1 : 15. In summary, although there are few reports on the adsorption of CO_2_ by adsorption materials based on coal gangue, the existing studies are sufficient to show the huge application potential in this field. In addition, the application of modified coal gangue in the field of greenhouse gas reduction still faces numerous challenges. For instance, the synthesis cost and process complexity which are involved in the pre-treatment and conversion processes of coal gangue. Additionally, although the research has achieved promising experimental results at the laboratory stage, there is still a long way to go from laboratory research to industrial-scale application. This requires coal gangue-based adsorbents to overcome various technical barriers, including large-scale production, cost control, and operational stability.

### 3.3 Application in developing catalysts

Natural kaolinite,^69^ pyrite under an inert atmosphere,^70^ and non-metallic carbon-based materials^71^ have been widely studied and proven effective in the catalytic degradation of organic pollutants. Due to its diverse composition (kaolinite typically accounting for 50% to 70%, followed by 20% to 30% quartz, and the remaining 10% to 20% consisting of carbonaceous materials and other minerals), coal gangue can be considered a potential catalyst. Li *et al.* developed a novel coal gangue-based persulfate catalytic material (GCM-ZnCl_2_) that effectively activates peroxymonosulfate (PMS) with a degradation rate of 82.6% within 25 minutes. The material was prepared from coal gangue, crushed and grinded to a certain particle size, then mixed with ZnCl_2_ evenly, before finally calcining the mixture at 750 °C for 90 minutes under an inert atmosphere.^72^ Zhang *et al.* designed and synthesized a series of metal-free, green, and sustainable hybrid catalysts (CN-CGs) based on solid waste coal gangue and graphitic carbon nitride (g-C_3_N_4_, CN), these catalysts were capable of removing 90% of bisphenol A (BPA) within 30 minutes, with a total organic carbon (TOC) removal efficiency of 80%.^73^ In addition to its application in the catalytic degradation of organic pollutants, coal gangue can also be used in catalytic cracking processes. Wang *et al.* synthesized fluid catalytic cracking (FCC) catalysts with higher catalytic activity for heavy oil cracking by activating coal gangue into highly reactive silica and alumina species using a green and energy-efficient self-combustion–depolymerization (SCD) method.^74^ Du *et al.* prepared porous coal char-based catalysts using coal gangue with a high metallic content and lignite through a simple pyrolysis method under a CO_2_ atmosphere for biomass tar decomposition. The exposure of metals on the catalyst surface facilitated efficient contact with the reactants, achieving a biomass pyrolysis tar conversion efficiency of 91.2% and a total syngas yield of 516 mL g^−1^.^75^

**Fig. 8:**
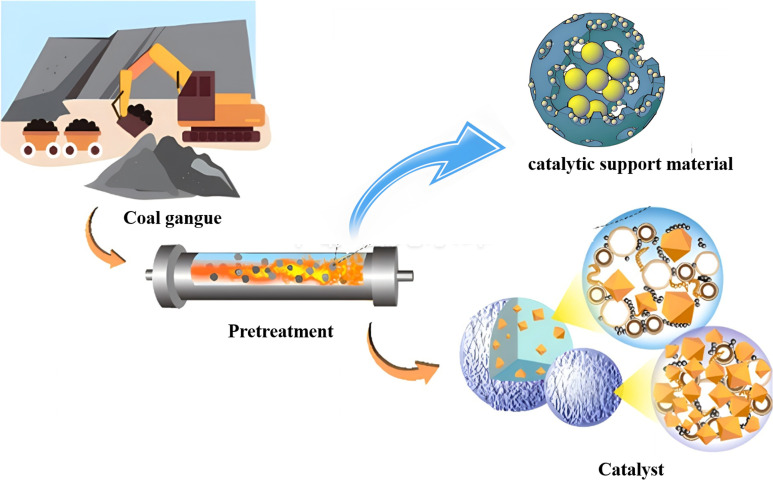
Application of coal gangue in catalytic applications.^74^

As shown in Fig. 8, the pre-treated coal gangue, in addition to being possible to convert it into a catalyst, has an inherent composition that allows it to be considered a potential catalytic carrier. Lu *et al.* applied acid-pretreated coal gangue as a support for a Ni-based catalyst in the catalytic reforming of toluene, achieving high toluene conversion and a significant H_2_ yield.^76^ Sun *et al.* used dual-activated coal gangue as the support for preparing Ni-based catalysts, which were effective in catalyzing the hydrogenation of dioctyl phthalate to produce di(2-ethylhexyl)hexahydro-phthalate, achieving a conversion rate of 99.9%. The Ni/ZMC-1 catalyst demonstrated the ability to be recycled up to 8 times with negligible loss of initial activity.^77^ Therefore, catalysts and carrier catalysts synthesized from coal gangue as raw materials exhibit significant catalytic efficiency both in the photocatalytic oxidation of organic compounds and in catalytic cracking technologies. Furthermore, the development and innovation of coal gangue-based hierarchical porous catalysts not only address the challenges in coal gangue disposal but also create high-performance, high-value new catalysts, enhancing the quality and efficiency of the catalytic application process. However, the durability of the catalyst during its use and the poisoning effect of heavy metals in coal gangue on the catalyst need to be given special consideration.

### 3.4 Applications in other areas

In addition to its extensive applications in the environmental field, coal gangue is also widely used in underground filling, the energy industry, as a building material, and in the high-value preparation of aluminum/silicon products, as shown in Fig. 1. For example, in the field of underground filling, the preparation of paste backfill materials involves varying proportions of water, cement, fly ash, and coal gangue. Tests on slump and the hydraulic–mechanical properties, such as segregation and water bleeding ratios, demonstrated the effectiveness of these materials in mitigating surface subsidence.^78^ Research has shown that optimizing the grain-size distribution and structure can enhance the mechanical performance of backfill materials.^79^ In the energy industry, as coal gangue possesses a certain carbon content and other combustible materials, the energy released during its combustion can be harnessed for power generation and heating applications. After more than 30 years of development, by 2022, China utilized 35 million tons of coal gangue for power generation, accounting for approximately 6% of its total utilization. Currently, China has over 120 coal gangue power plants with an installed capacity of 1.84 million kW, producing an annual electricity output of 8.7 billion kW h.^80^ In building applications coal gangue is used because it is rich in clay minerals, with SiO_2_, Al_2_O_3_, and Fe_2_O_3_ constituting more than 80% of its composition, making it a potential cement material. Using red mud and coal gangue to replace 50% of the raw materials for producing cementitious materials has been proven to be feasible.^81^ The inherent cementitious capability of fresh coal gangue is low. However, its activity can be significantly enhanced through mechanical and subsequent thermal activation, which increases its specific surface area and improves the compressive and flexural strength of the resulting gangue cement.^82^ In the field of high-value products, due to the significant presence of valuable elements in coal gangue, it has been promoted for the production of high value-added products such as aluminum-, silicon-, and carbon-based chemicals, and water glass. When using coal gangue to produce alumina and silicon, the Al_2_O_3_ and SiO_2_ contents should be greater than 35% and 30%, respectively. However, extracting aluminum oxide and silicon dioxide directly from coal gangue is challenging due to their stable crystalline forms. Pre-treatment typically involves acid leaching and alkaline leaching, with acid leaching being more widely used due to its simplicity and high efficiency.^2^

## 4. Challenges and suggestions

### 4.1 Challenges

Despite significant advancements in the methods and applications for coal gangue management, there remain development bottlenecks and challenges, the most critical being various technical limitations in the deep processing stage. Currently, the primary challenges in the resource utilization of coal gangue may include the following aspects. (1) High capital and technological costs: due to the immaturity of the technology, and the high cost and low-quality of the products, the competitiveness of coal gangue products is weak, failing to attract sufficient investment to promote its further development. The production process of coal gangue products is more complicated than ordinary similar products. For, example, the zeolites synthesized from coal gangue must be made in a high-temperature and strongly alkaline environment, leading to much higher costs compared to conventional zeolites. The treatment of coal gangue requires substantial capital and technological investment. The by-products of comprehensive coal gangue utilization often have low added value, resulting in high costs and low profits, which complicates their application and promotion. In the case of high-value-added products, the high production costs and complex processes mean that most remain at the experimental stage, with few breakthroughs, which suppresses enterprise enthusiasm and creates a vicious cycle. (2) The lack of unified standards: notably, coal gangue is utilized differently depending on the different coal gangue properties and the requirement of the products. However, there is no rational arrangement for the comprehensive utilization of coal gangue tailored to its properties that considers various utilization alternatives. The absence of strict project approval for coal gangue utilization leads to project stagnation or abandonment due to infeasibility. Furthermore, the lack of tracking management during the production process can result in substandard products, equipment damage, and environmental pollution, which collectively impede the advancement of coal gangue recycling. In China, for example, although the government has introduced a series of policies and regulations for the comprehensive utilization of coal gangue, there are no national standards for the treatment and utilization of coal gangue at the technical level. Consequently, this results in varying local standards and requirements for management, leading to potential risks in governance. (3) Residual waste management needs improvement: apart from the traditional utilization of coal gangue, like filling in the mining area, the production of building materials and power generation, a number of reports on high-value-added products provide a significant opportunity for coal gangue utilization, such as the preparation of adsorbents, and the separation and recovery of valuable elements. However, the management of residual waste remains challenging. In particular, the use of numerous chemical reagents during processing can lead to secondary pollution if not managed properly, potentially transforming solid waste into a situation where all three types of waste (solid, liquid, and gaseous) coexist, posing risks of secondary environmental contamination. (4) Recycling and utilization levels need improvement: the technological barriers are widely accepted as the pressing problem in the utilization of coal gangue. Despite the rich resources contained in coal gangue, current recycling and utilization technologies still need significant advancement. The utilization of coal gangue on high-value-added products provide a promising opportunity, however, most of these studies are still in the laboratory research stage and have not been applied on a large scale in practice.

Consequently, the treatment and comprehensive utilization of coal gangue present long-term and challenging tasks. Addressing these challenges requires sustained and collaborative efforts from the government, enterprises, and research institutions. Future progress in this field will depend on the combined contributions of these stakeholders to overcome existing limitations and enhance the effectiveness of coal gangue resource utilization.

### 4.2 Suggestion and outlook

#### 4.2.1 Scientific utilization of coal gangue according to its location and properties

Traditional methods for coal gangue resource utilization in the environment primarily include soil applications, the preparation of adsorption materials and the development of catalysts. Various applications need coal gangue with different physicochemical properties and mineral compositions and have different environmental impacts. In addition, these methods often encounter off-site disposal issues, which is not well integrated with local conditions. Therefore, it is essential to align utilization methods with regional needs and coal quality characteristics while adhering to the principles of “scale, high value, and sustainability”. Furthermore, it is valuable to explore the scientific combination of various coal gangue applications to minimize the production of waste and mitigate environmental impacts. This approach aims to combine conventional uses with high-value applications, promoting more efficient, environmentally friendly, and sustainable coal gangue utilization, thereby contributing significantly to the sustainable development of both the economy and society.

#### 4.2.2 Establish a tracking management system

The presence of toxic elements in coal gangue poses a risk of secondary pollution, it is necessary to establish a scientific management system to ensure project plans are met and to assess implementation status. Regular sampling and monitoring of raw materials, products, and emissions at plants are crucial for implementing timely measures to prevent environmental pollution. For instance, when coal gangue is utilized for subsidence filling in mining areas and underground backfill, it is essential to monitor soil and groundwater to prevent pollution from harmful elements. Similarly, when coal gangue is used in brick production, it is important to detect sulfur content, toxic elements in the product, and harmful gas emissions from the brick-making process. Consequently, tracking the transformation of trace elements throughout the coal gangue utilization life cycle is vital for environmental protection.

#### 4.2.3 Utilization of coal gangue in the future

In terms of current directions for coal gangue utilization, developing new types of artificial soil from coal gangue can help alleviate pressure on soil resources in mining areas and expand available land, providing opportunities for further resource utilization. This method also addresses issues related to the excavation and transport of soil while effectively utilizing locally stockpiled coal gangue, resulting in multiple benefits. Furthermore, advancing other new technologies, such as the coupling of coal gangue with other coal-based solid waste extraction technologies and comprehensive elemental utilization techniques, is crucial for achieving high-value and large-scale coal gangue utilization.

## 5 Conclusion

The production of coal gangue will rise considerably due to the rapid increase in energy demand, indicating a considerable opportunity for the utilization of coal gangue. This review explores the properties of coal gangue and various methods for its resource utilization in environmental applications, with a particular focus on its applications in soil, adsorbents, and catalyst synthesis. Despite the benefits of coal gangue utilization, challenges such as high capital and technological costs, the lack of unified standards for practical utilization, and the need for improved residual waste management hinder its large-scale application. In particular, the issue of secondary pollution during the resource utilization process requires attention. Thus, the scientific utilization of coal gangue according to its location and properties, along with the establishment of a tracking system, will be of great significance for its efficient use.

Owing to its local availability and properties, coal gangue-based artificial soil demonstrates significant potential for large-scale resource utilization. Furthermore, advancing other new technologies, such as the coupling of coal gangue with other coal-based solid waste extraction technologies and comprehensive elemental utilization techniques are future areas. Nonetheless, the potential for secondary pollution from heavy metals contained in coal gangue, that impacts the surrounding environment, alongside the persistence of coal gangue-based soil, necessitates further investigation. The insights provided in this review aim to inspire future research and inform policy decisions, paving the way for broader coal gangue applications.

## Data availability

No primary research results, software or code have been included and no new data were generated or analysed as part of this review.

## Author contributions

Writing—original draft preparation, L. G.; writing—review and editing, L. B. and N. G.; method and visualization, Y. L. and K. X.; supervision and project administration, S. L.; funding acquisition, L. G. All authors have read and agreed to the published version of the manuscript.

## Conflicts of interest

There are no conflicts to declare.
